# Unilateral endogenous Candida endophthalmitis after extracorporeal shock wave lithotripsy for a renal stone in an immunocompetent woman: a case report

**DOI:** 10.1186/s12348-019-0172-0

**Published:** 2019-02-20

**Authors:** Mohammad Shirvani, Shahla Hosseini, Merhrnoosh Maalhagh, Mehrdad Afarid, Ali Mirza-Khanlooi, Sahar Mohaghegh

**Affiliations:** 1 Poostchi Ophthalmology Research Center, Shiraz University of Medical Sciences, Shiraz, Iran; 2 Shiraz University of Medical Sciences, Shiraz, Iran

## Abstract

**Purpose:**

To report a case of endogenous Candida endophthalmitis that may cause catastrophic sight-threatening outcomes, after extracorporeal shock wave lithotripsy (ESWL) in a healthy woman.

**Case presentation:**

A 32-year-old woman presented to the ophthalmology clinic with the symptom of blurred vision and floater in her right eye. She underwent ESWL for renal stone 1 week prior to her presentation. Fundus examination showed an elevated white lesion in para-fovea with fluffy border. Smear of diagnostic vitreous sampling showed pseudo-hypha and budding yeast. Systemic and immunologic and infectious workups were unremarkable. In our case, response to intravitreal and intravenous injection of amphotericin-B and oral fluconazole was dramatic. Six weeks after starting the treatment, infiltrative lesion completely disappeared. The authors review previous MEDLINE literature about this topic.

**Conclusion:**

Endogenous Candida endophthalmitis following renal stone lithotripsy is a serious and rare intraocular infection that may happen in healthy individuals without any risk factors.

## Introduction

Endophthalmitis is a severe vision-threatening ocular infection. Hematogenous spreading of microorganisms into the ocular circulation secondary to systemic infection specially in immunocompetent patients is a serious condition and includes 2–10% of all of endophthalmitis forms [1]. The most common organisms that cause endogenous fungal endophthalmitis are Candida, *Aspergillus*, and *Coccidiodes* [2]. Here, we report a rare case of endogenous Candida endophthalmitis (ECE) following an extracorporeal shock wave lithotripsy (ESWL) for a kidney stone in a healthy woman without any history of urinary tract infection (UTI) and obstruction.

## Case presentation

**Fig. 1:**
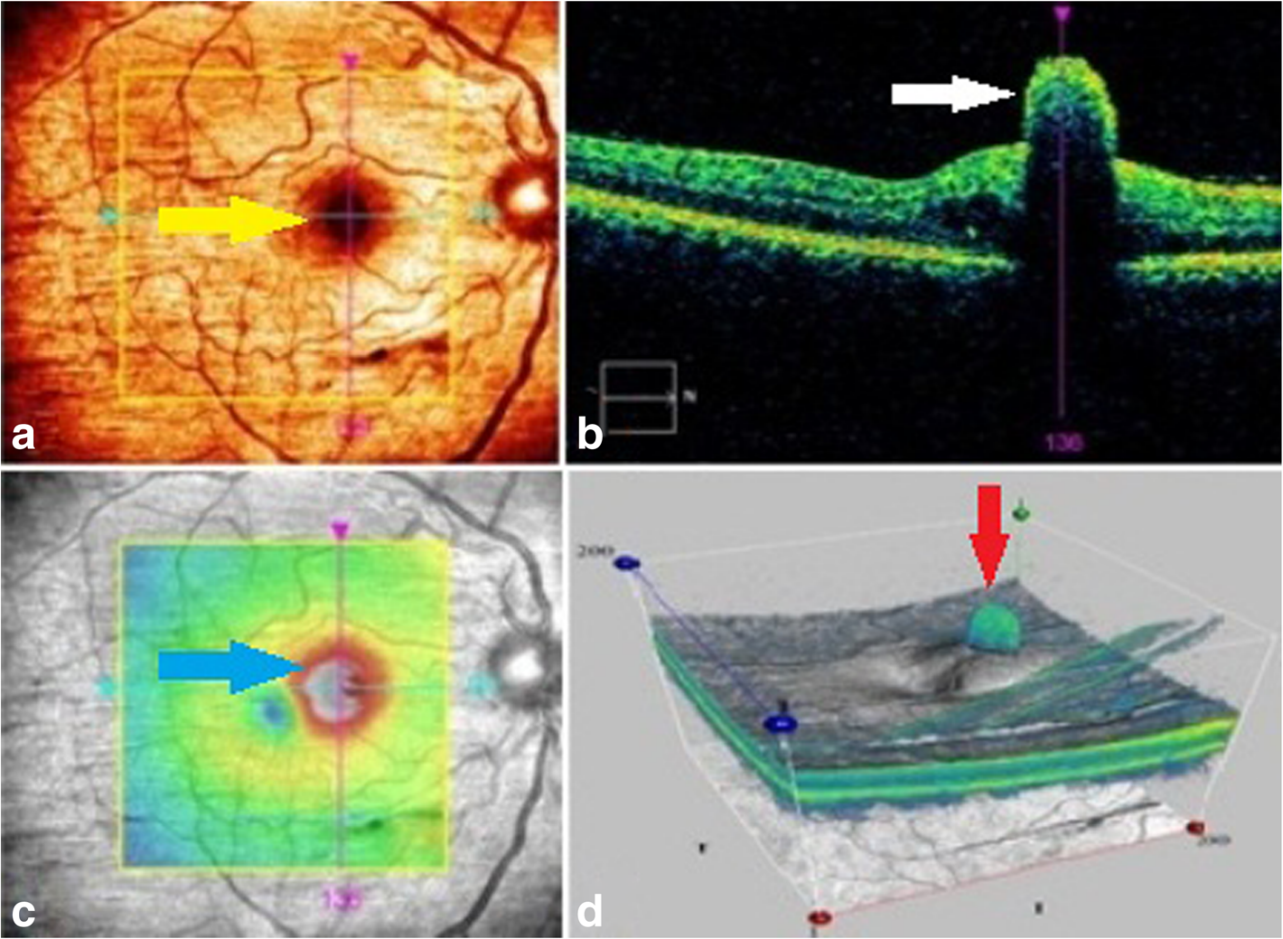
The right eye, at time of presentation: OCT fundus image reveals a para-fovea lesion (**a**), yellow arrow; spectral domain-OCT (SD-OCT) displays a hyper-reflective lesion in the vitreomacular interface and macular edema (**b**), white arrow; infrared fundus image shows a hot and elevated lesion (**c**), blue arrow; three-dimensional macular image reveals a retinal lesion with vertical expanding to vitreous cavity (**d**), red arrow

**Fig. 2:**
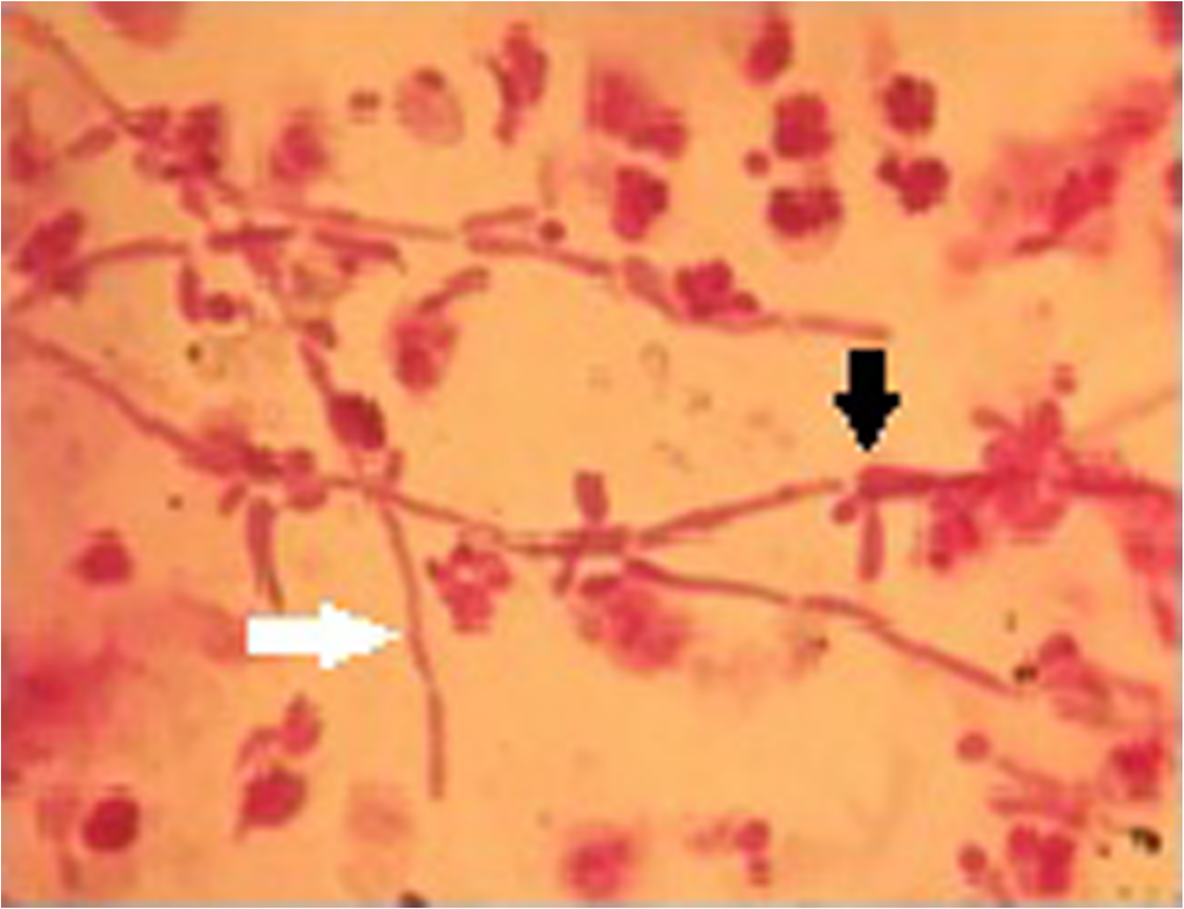
Vitreous sample smear with Gram staining reveals fungal spores and budding yeasts (black arrow) and pseudo-hypha and leukocyte infiltration (white arrow)

A 32-year-old woman presented at the ophthalmology clinic with chief complaints of floater and painless gradual decreased visual acuity in her right eye from 5 days earlier. There was no previous history of ocular surgery, trauma, systemic disease, and medication. There was a medical history of ESWL for a 12-mm right renal pelvis stone 1 week prior to her presentation. Pre- and post-operative urine culture was negative, and urine analysis was normal. In clinical examination, best-corrected visual acuity (BCVA) of the right eye decreased to line 20/40 of the Snellen chart. Left eye BCVA was 20/20. Intraocular pressure of both eyes was 15 mm/Hg. Right eye slit-lamp examination revealed conjunctival injection and + 1 cell in the anterior chamber. Also, fundus examination showed clear media with + 3 vitritis and an elevated white ball-like lesion with 1 disc diameter size, on para-fovea with fluffy border. The right eye macular optical coherence tomography (OCT) displayed a hyper-reflective lesion in the vitreomacular interface (Fig. 1). There was no remarkable sign in the examination of the left eye. Diagnostic vitreous tap was performed, and the sample was sent for smear and culture. The smear of the vitreous sample with Giemsa stain showed multiple fungal spores with budding yeast and fungal pseudo-hypha and leukocyte infiltration (Fig. 2). Cultures of the vitreous sample after 7 days were positive for *Candida albicans*.

The patient was admitted to the hospital; then, intravitreal injection of amphotericin-B (5 μg/0.5 ml) was performed and topical atropine 1% Q6hr, topical prednisolone acetate 1% Q4hr, topical ciprofloxacin 0.3% Q6hr, intravenous amphotericin-B 1 mg/kg/day, and oral fluconazole 100 mg Q12hr were started. Systemic workup including ANA, ANCA (P, C), AMA, VDRL, FT-ABS, Toxoplasma IgM and IgG, HBs Ag, HBc Ab, HCV Ab, HIV Ab, serology for *Borrelia* and *Bartonella*, Mantoux and interferon-γ test were all negative. Erythrocyte sedimentation rate, C-reactive protein, CBC and platelet, fasting blood glucose, AST and ALT, BUN, creatinine, ACE, IgG, IgM, and IgA level were within the normal limit. Peripheral blood smear, para-nasal sinus, and chest X-ray were normal.

**Fig. 3:**
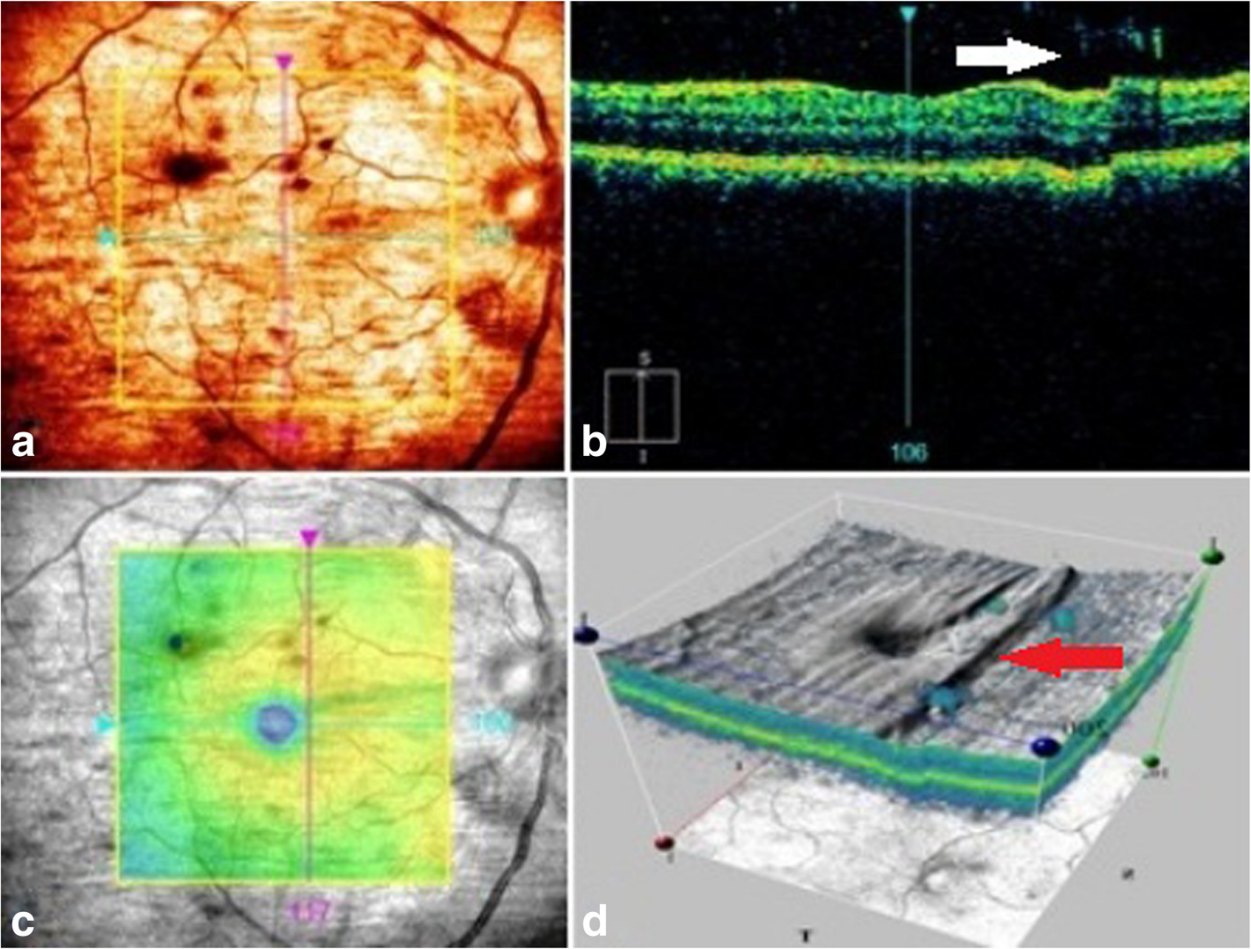
Regression of primary fungal lesion, 6 weeks after antifungal therapy. OCT fundus image reveals the disappearance of retinal lesion (**a**), white arrow; SD-OCT shows mild cellular debris on posterior vitreous and resolved macular edema (**b**); infrared fundus image displays complete disappearance of fungal lesion (**c**); three-dimensional macular image shows mild irregularity and local uneven of sensory retina under site of primary lesion (**d**), red arrow

After 72 h of treatment, BCVA improves to line 20/30 of the Snellen chart. The lesion size became smaller, and borders became clear and sharp. The patient was discharged, and treatment continued with oral fluconazole 100 mg Q12. After 6 weeks of initial treatment, the right eye BCVA was 20/20. The infiltrative lesion completely disappeared in fundus examination but a trace of vitritis was apparent. No signs of scar formation and traction of the retina were observable. The macular OCT revealed mild cellular debris in the site of the primary lesion (Fig. 3). There was no recurrence in a 2-year follow-up.

## Discussion

Endogenous Candida endophthalmitis is an uncommon, sight-threatening condition with poor visual acuity outcome [1–3]. Predisposing factors that have been reported for ECE include major gastrointestinal intervention, long-term systemic antibiotic use, recent hospitalization, indwelling catheters, candiduria, hyperalimination, prolonged intravenous line, hemodialysis, chronic liver disease, surgical abortion, neutropenia, immunomodulatory therapy, diabetes mellitus, hematopoietic stem cell and organ transplantation, alcoholism, intravenous drug abuse, and HIV [1–7].

Extracorporeal shock wave lithotripsy is a noninvasive modality that is wildly used for the treatment of some renal and ureteral stones. Predisposing conditions that have been reported for infectious complications of ESWL include pre-existing UTI; struvite and infected calculi; multiple, large (> 2 cm), and stag-horn stones; urinary tract anatomical abnormality; urinary obstruction; instrumentation; and stent-catheter in the kidney and urinary tracts [6–11]. In our case, ECE occurred as a complication of ESWL in a healthy young woman with no underlying factor. There are few case reports of ECE following renal and ureteral stone lithotripsy that all of them had predisposing conditions (Table 1) [5–7, 10, 11].

**Table 1:** Data of previous case reports of endogenous Candida endophthalmitis after renal stone lithotripsy since 2000–2018

Author	Age (sex)	Type of lithotripsy	Predisposing factor(s)	OD/OS	Treatment	Time of ocular presentation
Lavine [5]	48 (M)	ESWL with ureteral stent	Rheumatoid arthritis on oral prednisone, positive urine culture for *C. albicans*	OD	Intravitreal injection of amphotericin-B+ intravenous amphotericin-B+ systemic voriconazole	One month after ESWL
Yuan Z [6]	54 (M)	ESWL with double-J Stent placement	Alcoholic liver disease, multiple stones, positive urine culture for *C. albicans*	Bilateral	Topical natamycin and fluconazole + systemic voriconazole + bilateral pars plana vitrectomy + silicon oil injection and fluconazole irrigation	7 days after ESWL
Toshikuni [7]	69 (M)	ESWL with double-J Stent placement	Liver cirrhosis, bladder catheter, candiduria	Bilateral	Systemic and topical fluconazole	23 days after ESWL
Inn [10]	46 (M)	Trans-ureteral lithotripsy with ureteral stent	Diabetes mellitus, positive urine culture for *C. albicans*	OD	Intravitreal injection of amphotericin-B and voriconazole + intravenous amphotericin-B and voriconazole + oral voriconazole + 2 time pars plana vitrectomy	5 days after trans-urethral lithotripsy
Barbosa [11]	59 (F)	Decompressive nephrostomy	Obstructive pyelonephritis	Bilateral	Intravenous fluconazole + intravitreal voriconazole + pars plana vitrectomy	One month after nephrostomy

Administrations of prophylactic antibiotics for patients who undergo ESWL are controversial according to previous studies. In several studies, pre-operative antibiotics are recommended in patients with high-risk factors such as having instrument or stent at the time of ESWL, positive urine culture, infected and stag-horn stones, and recurrent UTI [7–9]. The importance of prophylactics antifungal administration for patients who undergo ESWL has not been investigated so far. Therefore, future study on this topic is recommended. In our case, the patient did not receive any prophylactic antibiotics or antifungal before and after ESWL.

Endogenous Candida endophthalmitis is usually diagnosed by typical ocular lesions in patients with predisposing factors. Thus, clinical diagnosis of this condition may become challenging in patients with no underlying factors or those who present with atypical ocular signs. Misdiagnosis of the condition or delay in initiation of treatment may lead to devastating visual outcome [1–4]. So, diagnostic vitreous tap or diagnostic vitrectomy is recommended for definitive diagnosis in suspicious conditions. Real-time polymerase chain reaction (RT-PCR) of the vitreous sample is a more sensitive and rapid method to diagnose the etiology of endogenous endophthalmitis [1, 2]. A recent diagnostic method is more recommended if it was available.

The prognosis of visual acuity in endogenous fungal endophthalmitis depended on the location of fungal involvement (central or peripheral) and aggregation, timely diagnosis, type of organisms, and severity of ocular involvement. It also depends on structural changes of the involved area such as scar formation, tractional fibrotic membrane, and retinal detachment [1–3, 6]. In this case report, the patient’s healthy immune system, rapid diagnosis, and timely initiation of antifungal agents promoted a good visual prognosis.

## Conclusion

Endogenous Candida endophthalmitis after renal stone lithotripsy is a rare and visual-threatening condition that may even occur in immunocompetent individuals with no preliminary risk factors. Timely diagnosis and rapid antifungal therapy would guarantee a good visual outcome.

## Abbreviations

BCVA: Best-corrected visual acuity
ECE: Endogenous Candida endophthalmitis
ESWL: Extracorporeal shock wave lithotripsy
OCT: Optical coherence tomography
RT-PCR: Real-time polymerase chain reaction
UTI: Urinary tract infection
